# Feasibility of a low-intensity intervention to improve antimicrobial use in outpatient settings

**DOI:** 10.1017/ash.2023.386

**Published:** 2023-09-29

**Authors:** Brigid Wilson, Sunah Song, Taissa Bej, Ukwen Akpoji, Corinne Kowal, Federico Perez, Robin Jump

## Abstract

**Background:** Overall, ~12% of outpatient visits result in an antibiotic prescription, and 30% of those prescriptions are inappropriate. Behavioral nudges help influence practitioner behavior. We hypothesized that peer comparison combined with a behavioral nudge (a patient alert letter) would influence prescribers to reduce antibiotic prescriptions and improve antimicrobial stewardship in the outpatient setting. We pilot-tested this intervention in outpatient primary care clinics associated with a large Veterans Affairs (VA) medical center. **Methods:** We conducted a clustered randomized controlled trial of 12 community-based outpatient clinics. All practitioners in the intervention arm received quarterly comparative feedback reports and, when indicated, quarterly patient alert letters. Comparative feedback reports gave personalized feedback about antibiotic prescriptions for upper respiratory tract infections, comparing the recipient’s antibiotic prescriptions to the average for all practitioners at the primary care clinics included in our study. Patient alert letters notified practitioners to patients in their panel with recently detected *Clostridioides difficile* or resistant organism and their antibiotic exposures. We assessed outpatient visits during the preintervention period (April–September 2020), the intervention period (October 2020–September 2021), and the postintervention period (October 2021–September 2022). A mixed-effects logistic regression model predicting antibiotic prescriptions compared the arms across these periods. **Results:** The outpatient populations observed in the intervention and control arms were similar during each phase of the study. Prior to the intervention, the average proportion of visits with an antibiotic prescription was lower among clinics in the intervention arm (1.4% vs 1.8% in control arm; *P* = .45). This difference broadened slightly during the intervention period (1.4% vs 2.1%, respectively; *P* = .03) and the postintervention period (1.3% vs 2.1%, respectively; *P* = .01) (Fig. 1). Throughout the study, clinics in the intervention arm typically used more doxycycline and azithromycin and less amoxicillin-clavulanate and sulfamethoxazole-trimethoprim compared to clinics in the control arm. (Fig. 2). In the 6-month preintervention period, which coincided with the early phase of the COVID-19 pandemic, antibiotic prescriptions in the intervention compared to control clinics were similar. During the intervention and postintervention periods, the proportion of visits with an antibiotic prescription remained steady for clinics in the intervention arm and increased for those in the control arm. These results suggest that this pilot study using a low-intensity intervention consisting of comparative feedback reports and patient alert letters was successful in influencing the antibiotic prescribing behavior of primary care clinicians practicing in community-based outpatient clinics affiliated with a VA medical center.

**Figure:**
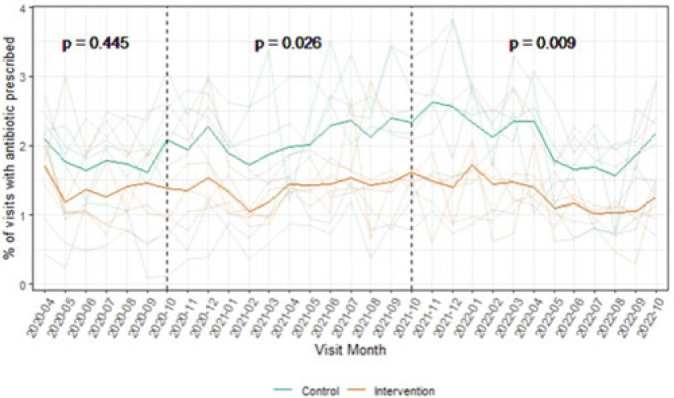

**Figure:**
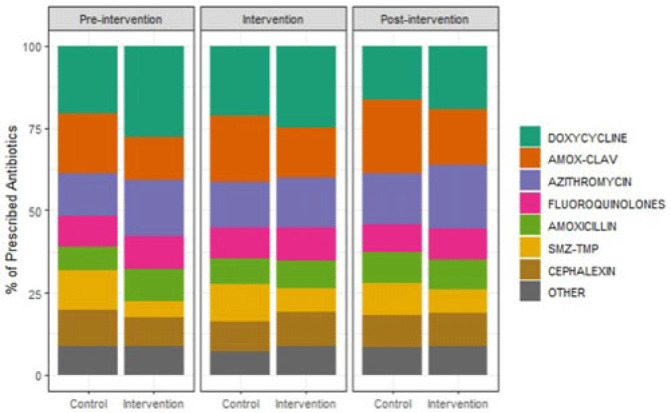

**Financial support:** This study was funded by Merck.

**Disclosures:** None

